# Pustular Eruption following COVID-19 Vaccination: A Narrative Case-Based Review

**DOI:** 10.3390/vaccines11081298

**Published:** 2023-07-29

**Authors:** Emmanouil Karampinis, Agoritsa Gravani, Polyxeni Gidarokosta, Dimitrios Petros Bogdanos, Angeliki-Viktoria Roussaki-Schulze, Efterpi Zafiriou

**Affiliations:** 1 Department of Dermatology, Faculty of Medicine, School of Health Sciences, University General Hospital of Larissa, University of Thessaly, 41110 Larissa, Greece; emankarampinis@uth.gr (E.K.); xeniagid@gmail.com (P.G.);; 2 Department of Rheumatology and Clinical Immunology, Faculty of Medicine, School of Health Sciences, University General Hospital of Larissa, University of Thessaly, 41110 Larissa, Greece

**Keywords:** COVID-19, vaccination, pustular eruption, pustular psoriasis, acute generalized exanthematous pustulosis, neutrophilic pustular eruption

## Abstract

From the beginning of public vaccinations until the relaxation of COVID-19 measures, many case reports, case series and case–control studies have been published indicating cutaneous side effects of COVID-19 vaccination. Post-vaccination pustular eruption was reported as well, with a challenging differential diagnosis between pustular psoriasis, AGEP (acute generalized exanthematous pustulosis) and neutrophil pustular eruptions. We report a case of 56-year-old woman presented with acute generalized pustular flare up culminated 5 days after the second dose of BNT162b2(Pfizer) vaccination. She was diagnosed with pustular psoriasis flare and due to the regulating role of IL-1 in pustular psoriasis and in the cytokine storm observed in cases of COVID-19 postvaccination inflammation; we decided to treat the patient with an IL-1 antagonist, subcutaneous anakinra (100 mg daily) along with acitretin. One week later, after anakinra withdrawal, she presented a pustular psoriasis flare and a 7-day anakinra re-administration led to a satisfactory improvement in the skin lesions. We also reviewed the medical literature and found 28 case reports with pustular eruption after the COVID-19 vaccination. We compared the patients reported, regarding sex, age, number of doses, post-vaccination period and vaccine brand, and compared those results with our patient. Finally, as indicated by our case and other cases with similarly treated pustular eruptions. targeted therapy to this cytokine imbalance such as anakinra (IL-1) antagonist can improve the clinical course of the patient.

## 1. Introduction

At present, the most efficient method for controlling and preventing epidemics, limiting the severity of the respective disease, reducing hospitalizations, and lowering the mortality rate is vaccination. COVID-19 vaccines including messenger RNA (mRNA) (Pfizer-BioNTech and Moderna) and viral vectors (Johnson & Johnson’s Janssen and AstraZeneca) were effective measures against COVID-19 pandemic [1]. The mRNA vaccines represented a ground-breaking approach that uses genetically modified RNA or DNA to generate viral spike proteins that stimulate the human immune system safely and effectively, while the viral vector category employs a genetically modified virus (adenovirus) to generate immunity. Additionally, other vaccine platforms have been developed, such as the inactivated virus vaccine Sinovac and the protein subunit vaccine Novavax [2] However, there are occasions that COVID-19 vaccinations have been associated with an extensive variety of dermatology diseases and have challenged the dermatology care and therapeutic management of the affected individuals [3,4].

Pustular skin eruption was a type of post-COVID-19 vaccination exanthem that was referred to in the medical literature and often included diagnosis of pustular psoriasis (new onset or flare) [5], acute generalized exanthematous pustulosis (AGEP) [6] and neutrophilic or eosinophilic pustular eruptions [7,8]. Generally, pustules are defined as localized accumulations of white blood cells and serous fluid that are contained within a circumscribed area and are the main or secondary clinical sign of many skin dermatoses [9]. Pustules are created through an anomalous buildup of white blood cells, sometimes with or without microorganisms and cellular waste, and can affect a follicle (folliculitis) [9].

The exact immunopathogenesis link that connects the immune reaction mediated by the vaccine and the pathophysiology of the aforementioned pustular diseases remains obscure. Vaccination is proven to lead to a systemic immune activation resembling a cytokine storm with the production of various pro-inflammatory cytokines that participate in the pathogenesis of pustular dermatosis [5]. In case of generalized pustular psoriasis, IL-1, along with IL-36, stimulates the expression of neutrophil chemokines, leading to neutrophil infiltration and pustule formation [10]. On the other hand, the production of IL-1 cytokines by human immune cells is stimulated by vaccines that lead to the induction of pro-inflammatory cytokines, regulating inflammation process [11]. Also, the association between COVID-19 vaccination and dermatology complications seem to be enhanced by environmental triggers such as vitamin D insufficiency [12].

Herein, we present a case of a patient that developed pustular psoriasis flare after the second dose of mRNA COVID-19 vaccine who was successfully treated with IL-1 antagonist (anakinra) and we attempt to critically review the published case reports and case series of COVID-19 vaccine-associated pustular eruptions to aid proper and accurate diagnosis and efficacious treatment.

## 2. Case Presentation

**Figure 1:**
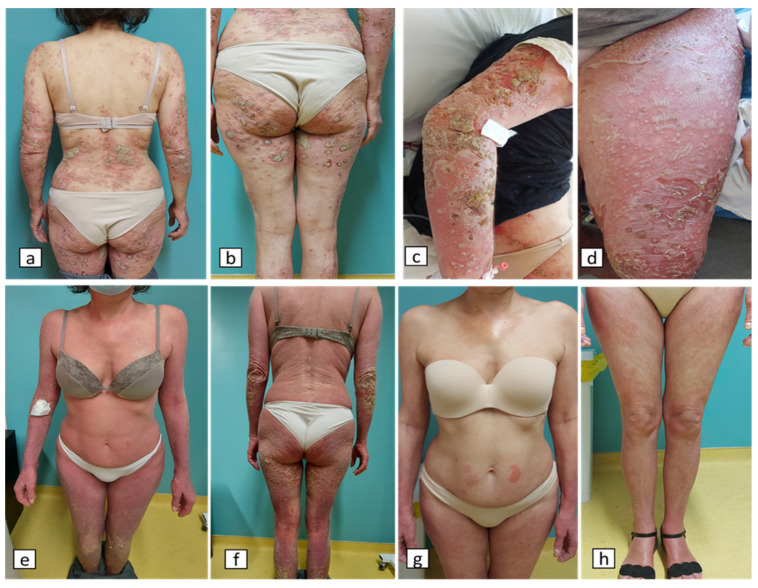
Images (**a**–**d**): the patient presented with painful skin patches accompanied with pinhead-sized sterile pustules on erythematous base and desquamation, as well as Nikolski positive sign with the lesions mainly located on the back, upper arms, abdomen area, thighs, soles and palms. Images (**e**–**h**): the patient presents with an improved skin clinical image, with the clinical lesions resolved by the tenth day after anakinra initiation.

**Figure 2:**
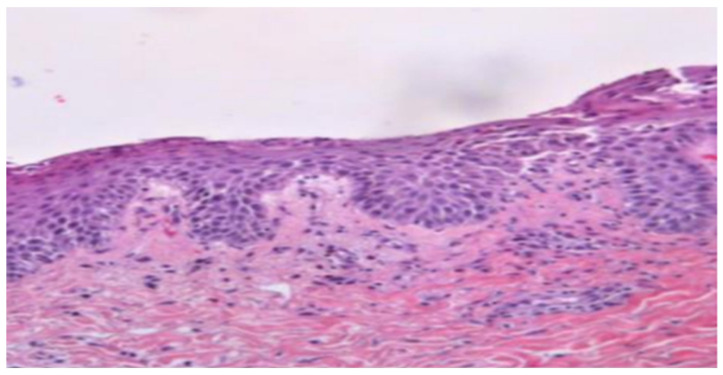
Histopathology of psoriatic lesions presenting with large Munro abscess and loss of granular layer and acanthosis.

A 56-year-old woman (BMI = 29 kg/m^2^) came to the Emergency Department of the University General Hospital of Larissa, Central Greece with acute generalized pustular flare up that occurred 5 days after the second dose of BNT162b2(Pfizer) vaccination. The prior four months, she was under treatment with methotrexate (10–15 mg/week) for plaque psoriasis control plus conventional filicine supplements. Three days after the first dose of the vaccination, the patient reported the appearance of small pustules on the abdomen and thighs. She was initially treated with topical corticosteroid preparations but with moderate response. The fifth day after the second dose of her vaccination, the patient presented with painful erythematous skin patches with pinhead-sized sterile pustules, mainly located on the back, upper arms, abdomen area, thighs, soles and palms (Figure 1), as well as systemic symptoms such as malaise, fever and muscle pain combined with extremely high CRP blood levels. Exfoliation and erythema were observed during the clinical examination and a skin biopsy was performed. Histopathology revealed Munro microabscesses, particularly in the upper layers of the epidermis, acanthosis and hyperkeratosis, as well as regions with intraepidermal acantholysis, skin swelling with capillary dilation and diffuse presence of lymphocytes. The above findings suggest that the diagnosis is generalized pustular psoriasis (Figure 2). A gene test for IL36RN and CARD14 genes, whose variants are gene-contributors to pustular dermatosis [13], was proposed, but due to patient’s personal refutation was not performed. At first, the patient was treated with antibiotic treatment and acitretin at a dose of 35 mg once daily for 7 days with no response. Due to the common pathway of IL-1 that shares the pathophysiology of pustular psoriasis as well as the inflammatory process and/or the unstable cytokine production triggered by COVID-19 vaccination [11,12], anakinra was initiated with a daily subcutaneous injection of 100 mg. Administration of acitretin at a dose of 25mg/day was continued. Pustules and the accompanied erythematous plaques resolved the tenth day after anakinra initiation (Figure 1). One week later after stopping anakinra, she presented a pustular psoriasis flare and anakinra was re-administered at a dose of 100 mg subcutaneous for 7 days in combination with a lower dose of acitretin (10 mg/day). After 7 days, the skin lesions improved remarkably. A dose reduction and a day-to-day administration of anakinra was followed while, at the same time, treatment with guselkumab, a human IgG1λ monoclonal antibody (mAb) against IL-23, was started with a dose of 100 mg, for control of the plaque psoriasis disease. An IL-17 inhibitor was not preferred due to previous failure of the specific drug category to treat the psoriasis disease in this patient. The medication scheme preserved clinical remission. The patient, despite the recent vaccination, had COVID-19 infection after three months with mild clinical course that did not also affect the course of the patient skin disease.

## 3. Methods

Due to the interesting nature of the case report and the interest of dermatology research in the skin manifestations after COVID-19 vaccinations, we performed research for pustular dermatoses related to COVID-19 vaccination. The list for pustular eruptions is extent. Therefore, we used the classification of Mengesha YM et al. [9] that separated pustular eruptions to generalized pustular dermatoses, pustular drug eruptions and localized pustular eruptions. Neutrophilic pustular dermatoses were added in this list as generalized or localized pustular eruption regarding their distribution (Table 1). Our inclusion criteria were a mainly pustular presentation of a skin disorder and a pustular eruption that appeared less than 12 weeks after vaccination to avoid confounders. Patients with previous COVID-19 infections or cases with neonates and children were excluded. Also, skin disorders with appearance of pustules as an atypical sign of the skin disorder or pustular eruptions with infectious etiology and not directly associated with vaccination were not studied. The search included PubMed articles published until the end of April 2023 and was based on terms such as “Acute Generalized Exanthematous Pustulosis” OR “pustular psoriasis” OR “pustular eruption” OR “pustules” AND “COVID-19 vaccine” OR “SARS-CoV-2 vaccine”. We used every term referred to the right column of Table 1 with COVID-19 vaccination terms.

## 4. Results

We describe the primary outcomes of our study, which include the PRISMA flow and tables outlining the patient characteristics of COVID-19 vaccination associated pustular skin diseases that we identified. Amongst them, we found cases of generalized pustular dermatosis, pustular drug eruption and localized pustular eruptions categories (Table 1). Also, as flare, we identified an exacerbation originating from a subtype of the same disease as pustular psoriasis occurring in a patient with plaque psoriasis.

A total of 63 articles were identified by our search strategy from Pubmed. From this, 35 articles were subsequently excluded due to ineligibility and therefore our narrative review included 28 case reports.

We found 16 patients reporting a generalized pustular eruption after COVID-19 vaccination (Table 2). Most of the cases were pustular psoriasis exacerbation (*n* = 10) occurring with a mean of 4-5 days after vaccination. Sex distribution was male dominant (*n* = 7) and age range was 18 to 72 years old. The most prevalent vaccine type regarding pustular psoriasis flare was Pfizer (*n* = 7). This flare occurred in most of the times after the first dose (*n* = 6). The new-onset pustular psoriasis eruptions were less (*n* = 3) than the flares. Neutrophilic generalized pustular dermatosis was also observed (*n* = 3) with more severe clinical presentations. Worth mentioning was the case of [14], which did not have a definite diagnosis as AGEP or a pustular psoriasis flare, so it was not included in our aforementioned results while it was included in the review of Wu et al. [5] as a pustular psoriasis flare.

Four of the cases [15,16,17,18] were confirmed with histopathology examination revealing AGEP diagnosis with the presence of discrete and confluent pustules, contrary to the pustular psoriasis Kogoj’s spongiform pustules, which are the accumulations of neutrophils under the stratum corneum. AGEP was combined with DRESS in two of the reported cases. Male dominance (4 out of 6) was observed in AGEP cases, and the age ranged from 27 to 74 years old. We noted cases reporting an eruption beginning in the first day after vaccination and others occurring after weeks. Also, a variety of vaccines brands associated with AGEP was observed (Table 2 and Table 3).

**Table 2:** Presentation of generalized pustular eruptions occurring after COVID-19 vaccination.

Study	Disease	Sex	Age	Clinical Outcome	Days after Vaccination	Vaccine	Dose of Vaccination	Treatment Strategy
[19]	Pustular psoriasis (Flare)	M	72	Generalized pustular psoriasis	4 days	Sinovac	First	Acitretin and intravenous Infliximab
[20]	Pustular psoriasis (Flare)	F	22	Generalized pustular psoriasis	3 days	Pfizer	First	Not Mentioned
[21]	Pustular psoriasis (Flare)	M	21	Generalized pustular psoriasis and erythroderma	4 days	Pfizer	Second	Anti-TNF biologic agent
[22]	Pustular psoriasis (Flare)	F	60	Generalized pustular psoriasis and erythroderma	8 days	Pfizer	Second	Oral etretinate
[22]	Pustular psoriasis (Flare)	M	18	Generalized pustular psoriasis and erythroderma	7 days	Pfizer	First	Cyclosporine and secukinumab
[23]	Pustular psoriasis (Flare)	M	40	Generalized pustular psoriasis and erythroderma	5 days	Pfizer	First	Cyclosporine and infliximab
[24]	Pustular psoriasis (Flare)	F	47	Generalized pustular psoriasis and erythroderma	10 days	Pfizer	Second	Risankizumab
[25]	Pustular psoriasis (Flare)	M	65	Generalized pustular psoriasis and erythroderma with systemic capillary leak syndrome	12 days	Pfizer	Second	secukinumab
[26]	Pustular psoriasis (Flare)	M	53	Scales and pustules	4 days	BBIBP-CorV	First	Neotigason and systemic steroids.
[27]	Pustular psoriasis (Flare)	M	21	Generalized pustular psoriasis	4 days	covaxin	First	Acitretin
[28]	Pustular psoriasis (New onset)	F	64	Erythema, scales, annular pustular psoriasis	NM	Pfizer	First	Methotrexate
[29]	Pustular psoriasis (New onset)	F	66	Erythema and pustules	21 days	AstraZeneca	First	Acitretin
[30]	Pustular psoriasis (New onset)	M	20	Erythema, pustules, crusts	4 days	Pfizer	First	Acitretin
[31]	Sneddon-Wilkinson	M	21	Vesiculopustular eruption and crust	8 days	Moderna	Second	Prednisone
[32]	Sweet-syndrome (pustular eruption)	M	77	Pustular eruption, encephalitis, myoclonus	2 days	Moderna	First	Prednisolone
[7]	Neutrophilic pustular eruption	M	32	Erythematous papulonodules and pustules and aphthous stomatitis with painful genital erosions	5 days	ChAdOx1	First	Dapsone and antibiotic treatment

**Table 3:** Presentation of acute generalized exanthematous pustulosis after COVID-19 vaccination.

Study	Disease Type	Sex	Age	Clinical Outcome	Days after Vaccination	Vaccine Type	Dose of Vaccination	Treatment Strategy
[15]	AGEP (With DRESS characteristics in follow-up)	F	40	Pustules and suberythema	7 and 11 weeks after first and second dose	First and Second	Pfizer	Prednisolone
[16]	AGEP-DRESS	M	43	Erythematous edematous papules, plaques and pustules	Hours (1 day)	Second	Moderna	Prednisolone
[33]	AGEP	F	32	Erythema and pustules	3 weeks	First	ChAdOx1	Topical treatment
[34]	AGEP	M	27	Erythema and pustules	Hours (1 day)	First	Moderna	Not mentioned
[17]	AGEP	M	27	Erythema and pustules	8 days	First	Moderna	Prednisolone
[18]	AGEP	M	74	Erythematous plaques and pustules	1 day	First	Janssen Ad26.COV2.S	Prednisolone and topical treatment

Regarding localized pustular dermatosis after COVID-19 vaccination (Table 4), we observed 9 patients in medical literature (Palmoplantar pustulosis *n* = 3, rosacea-pustulosis *n* = 2, facial pustular neutrophilic eruption *n* = 2, eosinophilic pustular folliculitis *n* = 1 and acute localized pustulosis *n* = 1). Although the categorization of facial pustular neutrophilic eruption in the pustular dermatosis list is unclear, we considered that disease in the localized pustular dermatosis due to its face-specific distribution. Amongst the patients, sex was almost equally distributed (Male:4 Female:5) and the age range was 38 to 80 years old. Most of the eruptions occurred the fourth or fifth day after the vaccination, with two cases occurring from the same article referring a postvaccination period of one month [35]. The responsible vaccine types were the Pfizer (*n* = 5) and Moderna (*n* = 3) vaccines. A specific dose (first or second) did not seem to contribute more to the development of such dermatosis. The patients, apart from the pustules, presented with the classical characteristics of the respective disease, for example erythema in rosacea-like pustular eruptions, and were treated with the classical disease-specific therapy. Half of the cases (4 out of 8) were new-onset skin manifestations, while palmoplantar psoriasis was due to exacerbation of a psoriasis subtype. Interestingly, an acne vulgaris flare was not detected. Worth mentioning is that, despite the localized distribution of pustular eruption, there are diseases with different pathophysiology origins and therefore occlusive conclusions cannot be drawn. Also, Table 5 summarizes the overall neutrophilic dermatoses observed in the study.

**Figure 3:**
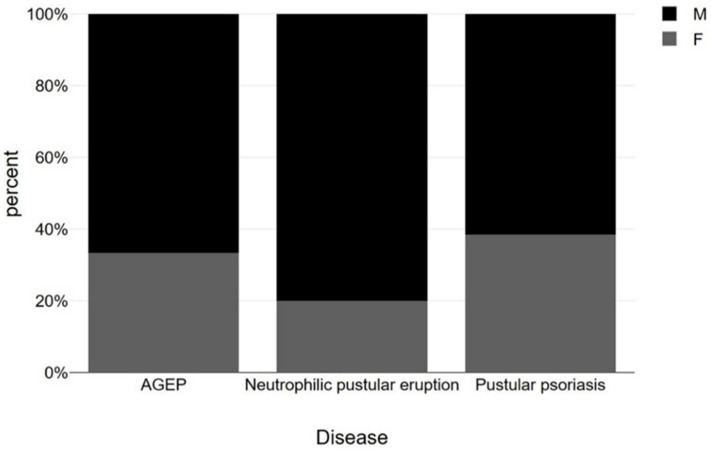
Presentation of sex distribution (using chi-square test) among AGEP, neutrophilic pustular eruption and pustular psoriasis cases occurred after COVID-19 vaccination (M: Male, F: Female) (*p* = 0.69).

**Figure 4:**
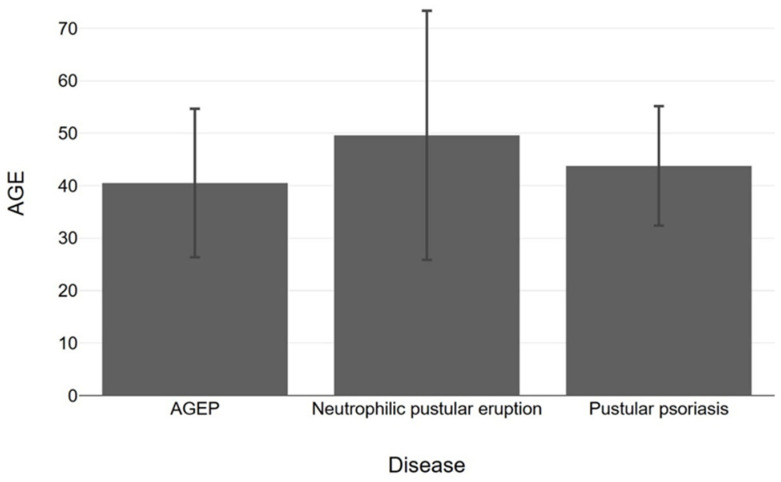
Presentation of age bars with 95% confidence interval (using *t*-test) amongst AGEP, neutrophilic pustular eruption and pustular psoriasis cases occurred after COVID-19 vaccination (*p* = 0.78).

**Figure 5:**
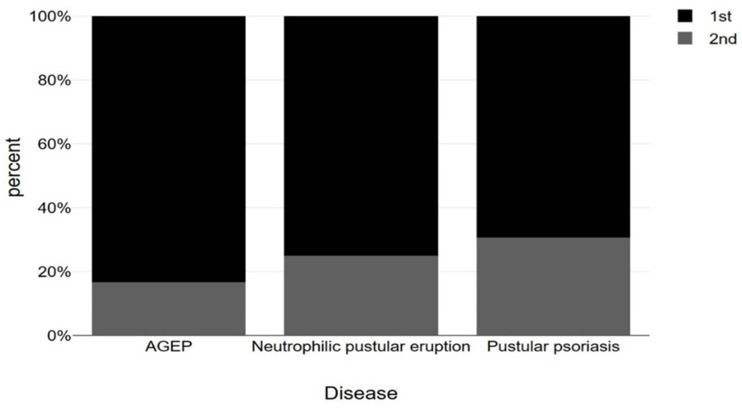
**Figure 5** Presentation of first vs. second dose of vaccination (using chi-square test) associated with AGEP, neutrophilic pustular eruption and pustular psoriasis cases (*p* = 0.68).

**Figure 6:**
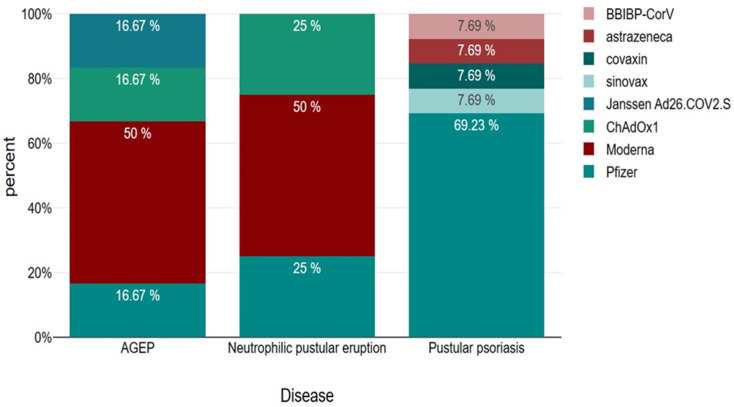
Presentation of vaccine distribution percentages amongst AGEP, neutrophilic pustular eruption and pustular psoriasis category cases.

Below are presented bar charts that compare sex, age, type of dose and vaccine brand in association with AGEP, pustular psoriasis and neutrophilic pustular dermatosis. Worth mentioning is the dominance of male sex in the disease cases, the prevalence of first-dose causality and Pfizer as the main type of vaccine causing pustular disease, especially in the case of pustular psoriasis (Figure 3, Figure 4, Figure 5 and Figure 6). However, statistical analysis (normal distribution proved by Shapiro–Wilk test, chi-square distribution test and ANOVA test) of the aforementioned case reports regarding sex distribution (*p* = 0.69), age (*p* = 0.78) and dose (*p* = 0.68) revealed no statistical importance, mainly due to a small amount of patients.

## 5. Discussion

In the beginning of the public vaccinations, the study of the cutaneous side effects of COVID-19 vaccination was limited, with only a few studies to have a large number of patients and focus on different dermatology conditions [40]. As time passed, case reports, case series and case–control studies from all over the world were published, revealing new post-vaccination disease occurrence or disease flare up. Also, third parameters in the relation between vaccination and dermatology disease were studied as environmental triggers [12]. All these aforementioned and the upcoming studies give a more complete image of the COVID-19 vaccination and its impact on dermatology. Also, more detailed data are revealed by studies on the immunology modifications that vaccinations can cause, creating a connecting link to understand the overlapping pathophysiologies. Pustular dermatoses were not the most frequent eruption occurring after vaccination; however, they challenged the dermatology practice due to the differential diagnosis between AGEP, neutrophilic pustular eruption and pustular psoriasis.

AGEP has a challenging differential diagnosis, which depends on both clinical and histopathology characteristics [6]. Cases reported in the review tried to assess the possibility of a pustular eruption connected with COVID-19 vaccination using the Naranjo score as a possible drug reaction [30]. Also, four of the cases based the diagnosis of AGEP on a histopathology report [15,16,17,18]. Contrary to AGEP, pustular psoriasis patients are more likely to have a medical history of plaque psoriasis, psoriasis comorbidities, presence of scales in clinical presentation and a histopathology report of spongiosis, macro and micro-pustules and psoriasis histopathology elements such as acanthosis [6]. Interestingly, one of the post-COVID-19 vaccination AGEP patients reported scalp psoriasis, which indicates that the prior medical history of psoriasis is not always a requirement [15]. The presence of subcorneal pustules in a histology would lead to the neutrophilic disease, subcorneal pustular dermatosis (Sneddon–Wilkinson disease) [6]. Also, the common coexistence of some diseases such as AGEP-DRESS can sometimes direct the diagnosis.

In addition to COVID-19 post-vaccination dermatoses, case reports regarding pustular diseases during the viral infection itself have been reported. Pustular psoriasis flare [41] as well as AGEP due to COVID-19 virus and mainly due to treatment used (hydroxychloroquine) against the disease were documented [41,42], and a surge of proinflammatory cytokines were supported to be the connecting link between COVID-19 disease and pustular eruption [41]. Also, as in our report, patients with previous skin disease such as psoriasis may are more susceptible to pustular eruption due to their modified immunity profile [41].

Even though our patient was female, the conclusive results of our review showed a male dominance in pustular eruption cases. This sex distribution was also showed by Megna et al., who studied psoriasis flare ups after COVID-19 vaccinations [42]. Since the number of patients analyzed was limited, it is not possible to reach reliable conclusions regarding male sex as a potential risk factor. Regarding the number of doses, both AGEP and pustular psoriasis occur after the first dose, with pustular psoriasis being the most frequent after the second dose of the vaccine amongst the pustular eruptions. This conclusion is in line with our case report. Also, our results indicate that pustular lesions appearing after administration of the Pfizer vaccine are more likely due to pustular psoriasis symptoms. This statement also correlates with our patient who developed pustules after the second dose of Pfizer vaccine.

An increasing number of studies are providing additional information regarding the immunological response of COVID-19 vaccines and attempting to elucidate the development of post-vaccine skin manifestations, including pustular eruptions. It is hypothesized that inflammatory cytokines release such as IL-1, IL-6 and TNF-a seem to contribute to the formation of pustule or general to skin flare [43]. The COVID-19 mRNA vaccine encodes the SARS-CoV-2 spike protein, which triggers IL-1β secretion in macrophages. The release of excessive amounts of IL-1 and IL-36 results in an inflammatory response in the skin, which leads to the development of pustules in pustular psoriasis [44]. Additionally, IL-6, compared with the other cytokines that are raised in pustular psoriasis, showed the strongest correlation with disease severity and systemic inflammation [45]. IL1 and IL6 are also noted to be high in the blood of patients with AGEP [46,47]. The dysregulated cytokine network triggered by the vaccine can lead to the imbalance of pro-inflammatory cytokines in neutrophilic dermatosis, leading to an overactive neutrophil response and persistent inflammation [48]. IL-6 can induce Th17 cells that, in turn, produce IL-17 and IL-22 that contribute to the proliferation of skin cells, triggering abnormal skin cell turnover and leading to the thickening and scaling of the skin seen in psoriatic lesions [44]. In the study by Heo et al., vaccination of ChAdOx1 nCoV-19 led to increased levels of proinflammatory cytokines, especially IL-6 and IL-1β, immediately after the first dose, confirming the inflammatory cytokine hypothesis of the pustular dermatoses [45]. The excessive and imbalanced cytokine production of vaccines has been confirmed in many postvaccination diseases and even vaccination-related death cases [49]. However, in the study of Heo et al., BNT162b2 did not led to a significant increase in proinflammatory cytokine levels after both the first and second doses, and consideration of the timing of most skin exacerbations places the inflammatory cytokine theory of postvaccination dermatosis in doubt, indicating other contributors for systemic reactions such as the vaccine immune responses [45]. This statement is also supported by the fact that some patients under biology treatment experience flare-ups [44]. The cytokine measurements of Heo et al., on the other hand, was performed on healthy individuals and not on psoriasis patients with different pro-existing cytokine profiles [45]. Therefore, new studies focusing on the cytokine modifications after COVID-19 vaccinations on psoriasis patients should be performed to understand the cytokine regulation after COVID-19 vaccination in these patients.

Contrary to the more classic treatment options used in the case reports discussed, in order to control pustular psoriasis flares in our patient we used an IL-1 antagonist, anakinra. Anakinra is a regimen that large studies have indicated does not show great efficacy in pustular psoriasis subtype [50] and it is used particularly in patients with revealed IL36RN pathogenic gene variants [51], as Anakinra blocks the action of IL-1 by binding to its receptor, which in healthy individuals is normally blocked by IL36 antagonist. Due to the patient’s denial, a genetic test was not applicable. We have chosen anakinra treatment, as IL-1 is proven to be the key regulator of inflammation after COVID vaccination [11] and it was also used to treat inflammatory disease bursts [52] as well as myocarditis cases [53] associated with cytokine storms caused by COVID-19 vaccines. Among the inflammatory disease flares were Still disease, psoriasis, and psoriasis arthritis with clinical and laboratory signs of inflammation and pro-inflammatory cytokine rise. Skin rash was noted in three out of four cases presented, and prednisone was accompanied by anakinra in the treatment plan. Anakinra was both used by subcutaneous and intravenous route of administration. In these cases [52], the follow up was without flare from 3 to 6 months under anakinra. In our case, anakinra’s temporary discontinuation led to the exacerbation of skin manifestations, further demonstrating the involvement of the Il-1 axis in the pathophysiology of vaccine-induced pustular psoriasis flares.

Our study presents some limitations. Firstly, there are categories presented in which the relevant studies and cases did not reach a statistically significant result, and secondly, only one medical database was used (Pubmed). Also, the concept of post-vaccination pustular eruption is new and therefore more case reports are anticipated to be published, giving new data that may not be included in the review.

## 6. Conclusions

Pustular eruption followed by vaccination can be a challenging diagnosis including pustular psoriasis, AGEP and neutrophilic dermatosis. Similarities in vaccine brands, post-vaccination flare timing and patient-related characteristics cannot further direct the diagnosis, making the clinical and histopathological disease features the main indicators. The cytokine imbalance caused by vaccination can possibly trigger the eruption, but studies on cytokine measurements on dermatology patients should be performed to confirm this hypothesis. Targeted therapy to this cytokine imbalance such as anakinra (IL-1) antagonist can improve the clinical course of the patient.

## Figures and Tables

**Table 1:** Presentation of the categories of pustular dermatoses as presented by Mengesha YM et al. [9] with the addition of neutrophilic pustular dermatoses.

Generalized pustular dermatosis	Generalized Pustular Psoriasis Reiter disease (Keratoderma blennorrhagicum) Subcorneal Pustular Dermatosis Generalized neutrophilic pustular eruptions
Pustular drug eruption	AGEP Acneiform eruption
Localized pustular eruption	Pustulosis palmaris and plantaris Acrodermatitis continua Acne vulgaris Rosacea Perioral dermatitis Folliculitis Localized neutrophilic pustular eruption Eosinophilic folliculitis

**Table 4:** Presentation of localized pustular eruption and COVID-19 vaccination associated case reports in the literature.

Study	Disease Type	Sex	Age	Clinical Outcome	Days after Vaccination	Vaccine Type	Dose of Vaccination	Treatment Strategy
[36]	Palmoplantar pustulosis (Flare)	M	57	NM	One month	Pfizer	NM	Oral acitetrin
[36]	Palmoplantar pustulosis (Flare)	F	63	NM	One month	Pfizer	NM	Oral acitetrin
[35]	Palmoplantar pustulosis (Flare)	M	60	Multiple pustules on the palms and soles	One week	Pfizer	Both first and second dose	Topical steroid application for two weeks
[37]	Rosacea (New-onset)	F	60	Erythema, telangiectasias and papulo-pustular eruption	4 days	Modena	First dose	Sun protection
[37]	Rosacea (NM onset)	F	47	Erythema, and papulo-pustular eruption	5 days	Pfizer	Second dose	Sun protection
[38]	Facial pustular neutrophilic eruption (New-onset)	M	50	Pustules and crust	4 days	Moderna	First dose	Antibiotic treatment and cortisol cream
[38]	Facial pustular neutrophilic eruption (New-onset)	M	80	Pustules, crust and erythema	5 days	Moderna	Second dose	Antibiotic treatment and calcineurin inhibitor cream
[8]	Eosinophilic pustular folliculitis (New-onset)	F	38	Pustules and mainly papules	2 days	Pfizer	First and Second dose	Topical corticosteroids
[39]	Acute localized pustulosis	F	43	Plaques and pustules	2 days	AstraZeneca	First	Topical treatment and oral prednisolone

**Table 5:** Summary of neutrophilic pustular eruptions after COVID-19 vaccination.

Study	Disease	Sex	Age	Clinical Outcome	Days after Vaccination	Vaccine	Dose of Vaccination	Treatment Strategy
[31]	Sneddon–Wilkinson	M	21	Vesiculopustular eruption and crust	4 days	Moderna	First dose	Antibiotic treatment and cortisol cream
[32]	Sweet-syndrome (pustular eruption)	M	77	Pustular eruption, encephalitis, myoclonus	5 days	Moderna	Second dose	Antibiotic treatment and calcineurin inhibitor cream
[33]	Neutrophilic pustular eruption	M	32	Erythematous papulonodules and pustules and aphthous stomatitis with painful genital erosions	5 days	ChAdOx1	First dose	Dapsone and antibiotic treatment
[38]	Facial pustular neutrophilic eruption (new-onset)	M	80	Pustules, crust and erythema	5 days	Moderna	Second dose	Antibiotic treatment and calcineurin inhibitor cream

## References

[1] Graña C., Ghosn L., Evrenoglou T., Jarde A., Minozzi S., Bergman H., Buckley B.S., Probyn K., Villanueva G., Henschke N. (2022). Efficacy and Safety of COVID-19 Vaccines. Cochrane Database Syst. Rev..

[2] Mascellino M.T., Di Timoteo F., De Angelis M., Oliva A. (2021). Overview of the Main Anti-SARS-CoV-2 Vaccines: Mechanism of Action, Efficacy and Safety. Infect. Drug Resist..

[3] Martora F., Battista T., Marasca C., Genco L., Fabbrocini G., Potestio L. (2022). Cutaneous Reactions Following COVID-19 Vaccination: A Review of the Current Literature. Clin. Cosmet. Investig. Dermatol..

[4] Shafie’ei M., Jamali M., Akbari Z., Sarvipour N., Ahmadzade M., Ahramiyanpour N. (2022). Cutaneous Adverse Reactions Following COVID-19 Vaccinations: A Systematic Review and Meta-Analysis. J. Cosmet. Dermatol..

[5] Wu P.-C., Huang I.-H., Wang C.-W., Tsai C.-C., Chung W.-H., Chen C.-B. (2022). New Onset and Exacerbations of Psoriasis Following COVID-19 Vaccines: A Systematic Review. Am. J. Clin. Dermatol..

[6] Parisi R., Shah H., Navarini A.A., Muehleisen B., Ziv M., Shear N.H., Dodiuk-Gad R.P. (2023). Acute Generalized Exanthematous Pustulosis: Clinical Features, Differential Diagnosis, and Management. Am. J. Clin. Dermatol..

[7] Bhargava A., Kharkar V., Mahajan S., Gole P. (2022). Neutrophilic Pustular Eruption with Behcet’s Like Illness Post Covid-19 Vaccination. Indian. Dermatol. Online J..

[8] Rikitake S., Kokubu H., Yamamoto B., Manabe T., Fujimoto N. (2022). Eosinophilic Pustular Folliculitis Developing at the Site of COVID-19 Vaccination. Clin. Exp. Dermatol..

[9] Mengesha Y.M., Bennett M.L. (2002). Pustular Skin Disorders. Am. J. Clin. Dermatol..

[10] Johnston A., Xing X., Wolterink L., Barnes D.H., Yin Z., Reingold L., Kahlenberg J.M., Harms P.W., Gudjonsson J.E. (2017). IL-1 and IL-36 Are Dominant Cytokines in Generalized Pustular Psoriasis. J. Allergy Clin. Immunol..

[11] Tahtinen S., Tong A.-J., Himmels P., Oh J., Paler-Martinez A., Kim L., Wichner S., Oei Y., McCarron M.J., Freund E.C. (2022). IL-1 and IL-1ra Are Key Regulators of the Inflammatory Response to RNA Vaccines. Nat. Immunol..

[12] Karampinis E., Goudouras G., Ntavari N., Bogdanos D.P., Roussaki-Schulze A.-V., Zafiriou E. (2023). Serum Vitamin D Levels Can Be Predictive of Psoriasis Flares up after COVID-19 Vaccination: A Retrospective Case Control Study. Front. Med..

[13] Mössner R., Wilsmann-Theis D., Oji V., Gkogkolou P., Löhr S., Schulz P., Körber A., Prinz J.C., Renner R., Schäkel K. (2018). The Genetic Basis for Most Patients with Pustular Skin Disease Remains Elusive. Br. J. Dermatol..

[14] Rouai M., Slimane M.B., Sassi W., Alaoui F., Chelly I., Mokni M. (2022). Pustular Rash Triggered by Pfizer-BioNTech COVID-19 Vaccination: A Case Report. Dermatol. Ther..

[15] Tay W.C., Lee J.S.S., Chong W.-S. (2022). Tozinameran (Pfizer-BioNTech COVID-19 Vaccine)-Induced AGEP-DRESS Syndrome. Ann. Acad. Med. Singap..

[16] Ikeda T., Yokoyama K., Kawakami T. (2022). Overlapping Acute Generalized Exanthematous Pustulosis Drug Reaction with Eosinophilia and Systemic Symptoms Induced by a Second Dose of the Moderna COVID-19 Vaccine. J. Dermatol..

[17] Mitri F., Toberer F., Enk A.H., Hartmann M. (2021). Acute Generalized Exanthematous Pustulosis in Close Temporal Association with mRNA-1273 Vaccine. Acta Derm. Venereol..

[18] Lospinoso K., Nichols C.S., Malachowski S.J., Mochel M.C., Nutan F. (2021). A Case of Severe Cutaneous Adverse Reaction Following Administration of the Janssen Ad26.COV2.S COVID-19 Vaccine. JAAD Case Rep..

[19] Onsun N., Kaya G., Işık B.G., Güneş B. (2021). A Generalized Pustular Psoriasis Flare after CoronaVac COVID-19 Vaccination: Case Report. Health Promot. Perspect..

[20] Durmaz I., Turkmen D., Altunisik N., Toplu S.A. (2022). Exacerbations of Generalized Pustular Psoriasis, Palmoplantar Psoriasis, and Psoriasis Vulgaris after MRNA COVID-19 Vaccine: A Report of Three Cases. Dermatol. Ther..

[21] Almeida R.O., Hanemann T., Peres F.L.X., Escobar G.F., Rangel Bonamigo R. (2022). Reactivation of Pustular Psoriasis Following MRNA Vaccination versus COVID-19 Infection: An Overlap?: Reply to “Generalized Pustular Psoriasis Following COVID-19” by Dadras MS et al.: Reply to “Generalized Pustular Psoriasis Following COVID-19” by Dadras MS et al. Dermatol. Ther..

[22] Tachibana K., Kawakami Y., Tokuda M., Sato S., Sugihara S., Miyake T., Sugiura K., Morizane S. (2022). Flare-up of generalized pustular psoriasis following Pfizer-BioNTech BNT162b2 mRNA COVID-19 vaccine: Two cases without mutations of *IL36RN* and *CARD14* genes. J. Dermatol..

[23] Perna D., Jones J., Schadt C.R. (2021). Acute Generalized Pustular Psoriasis Exacerbated by the COVID-19 Vaccine. JAAD Case Rep..

[24] Pavia G., Gargiulo L., Spinelli F., Avagliano J., Valenti M., Borroni R.G., Costanzo A., Narcisi A. (2022). Generalized Pustular Psoriasis Flare in a Patient Affected by Plaque Psoriasis after BNT162b2 mRNA COVID-19 Vaccine, Successfully Treated with Risankizumab. J. Eur. Acad. Dermatol. Venereol..

[25] Yatsuzuka K., Murakami M., Kuroo Y., Fukui M., Yoshida S., Muto J., Shiraishi K., Sayama K. (2022). Flare-up of Generalized Pustular Psoriasis Combined with Systemic Capillary Leak Syndrome after Coronavirus Disease 2019 MRNA Vaccination. J. Dermatol..

[26] Dayani D., Rokhafrouz H., Balighi K. (2023). Generalized Pustular Psoriasis Flare-Up after Both Doses of BBIBP-CorV Vaccination in a Patient under Adalimumab Treatment: A Case Report. Case Rep. Dermatol..

[27] Infimate D.L., Yumnam D., Galagali S.S., Kabi A., Kaeley N. (2022). Psoriasis Flare-Up After COVAXIN BBV152 Whole Virion Inactivated Vaccine. Cureus.

[28] Romagnuolo M., Pontini P., Muratori S., Marzano A.V., Moltrasio C. (2022). De Novo Annular Pustular Psoriasis Following MRNA COVID-19 Vaccine. J. Eur. Acad. Dermatol. Venereol..

[29] Elamin S., Hinds F., Tolland J. (2022). De Novo Generalized Pustular Psoriasis Following Oxford-AstraZeneca COVID-19 Vaccine. Clin. Exp. Dermatol..

[30] Frioui R., Chamli A., Zaouak A., Hlel I., Khanchel F., Fenniche S., Hammami H. (2022). A Case of New-Onset Acute Generalized Pustular Psoriasis Following Pfizer-BioNTech COVID-19 Vaccine. Dermatol. Ther..

[31] McCoy T., Shamsian D., Pan A., Sivamani R.K. (2023). Sneddon-Wilkinson Disease Following COVID-19 Vaccination. Dermatol. Online J..

[32] Torrealba-Acosta G., Martin J.C., Huttenbach Y., Garcia C.R., Sohail M.R., Agarwal S.K., Wasko C., Bershad E.M., Hirzallah M.I. (2021). Acute Encephalitis, Myoclonus and Sweet Syndrome after MRNA-1273 Vaccine. BMJ Case Rep..

[33] Kang S.-Y., Park S.-Y., Kim J.-H., Lee S.M., Lee S.P. (2021). COVID-19 Vaccine-Induced Acute Generalized Exanthematous Pustulosis. Korean J. Intern. Med..

[34] Agaronov A., Makdesi C., Hall C.S. (2021). Acute Generalized Exanthematous Pustulosis Induced by Moderna COVID-19 Messenger RNA Vaccine. JAAD Case Rep..

[35] Katsuie S., Nakamura K., Ogawa E., Arakura F., Okuyama R. (2022). Relapse of Palmoplantar Pustulosis Following COVID-19 Vaccination. Cureus.

[36] Piccolo V., Russo T., Mazzatenta C., Bassi A., Argenziano G., Cutrone M., Danielsson Darlington M.E.S., Grimalt R. (2022). COVID Vaccine-Induced Pustular Psoriasis in Patients with Previous Plaque Type Psoriasis. J. Eur. Acad. Dermatol. Venereol..

[37] Ciccarese G., Drago F., Rebora A., Parodi A. (2021). Two Cases of Papulo-Pustular Rosacea-like Eruptions Following COVID-19 Vaccinations. J. Eur. Acad. Dermatol. Venereol..

[38] Merrill E.D., Kashem S.W., Amerson E.H., Pincus L.B., Lang U.E., Shinkai K., Chang A.Y. (2021). Association of Facial Pustular Neutrophilic Eruption With Messenger RNA–1273 SARS-CoV-2 Vaccine. JAMA Dermatol..

[39] Wu R.-W., Lin T.-K. (2021). Oxford-AstraZeneca COVID-19 Vaccine-Induced Acute Localized Exanthematous Pustulosis. J. Dermatol..

[40] McMahon D.E., Amerson E., Rosenbach M., Lipoff J.B., Moustafa D., Tyagi A., Desai S.R., French L.E., Lim H.W., Thiers B.H. (2021). Cutaneous Reactions Reported after Moderna and Pfizer COVID-19 Vaccination: A Registry-Based Study of 414 Cases. J. Am. Acad. Dermatol..

[41] Miladi R., Janbakhsh A., Babazadeh A., Aryanian Z., Ebrahimpour S., Barary M., Sio T.T., Wollina U., Goldust M., Mohseni Afshar Z. (2021). Pustular Psoriasis Flare-up in a Patient with COVID-19. J. Cosmet. Dermatol..

[42] Ayatollahi A., Robati R.M., Kamyab K., Firooz A. (2020). Late-onset AGEP-like skin pustular eruption following COVID-19: A possible association. Dermatol. Ther..

[43] Schwartz R.A., Janniger C.K. (2020). Generalized Pustular Figurate Erythema: A Newly Delineated Severe Cutaneous Drug Reaction Linked with Hydroxychloroquine. Dermatol. Ther..

[44] Megna M., Potestio L., Gallo L., Caiazzo G., Ruggiero A., Fabbrocini G. (2022). Reply to “Psoriasis Exacerbation after COVID-19 Vaccination: Report of 14 Cases from a Single Centre” by Sotiriou E et al. J. Eur. Acad. Dermatol. Venereol..

[45] Heo J.Y., Seo Y.B., Kim E.J., Lee J., Kim Y.R., Yoon J.G., Noh J.Y., Cheong H.J., Kim W.J., Yoon S.-Y. (2022). COVID-19 Vaccine Type-Dependent Differences in Immunogenicity and Inflammatory Response: BNT162b2 and ChAdOx1 NCoV-19. Front. Immunol..

[46] Wang L., Pan J., Jin H. (2022). Profiling and Multivariate Analysis of Serum Cytokines in Patients with Generalized Pustular Psoriasis. Eur. J. Inflamm..

[47] Lalevée S., Audureau E., Riou A., Colin A., Anquetin M., Barau C., Valeyrie-Allanore L., Delfau-Larue M., Chosidow O., Wolkenstein P. (2019). Acute Generalized Exanthematous Pustulosis and Epidermal Necrolysis Differ in Innate Cytokine Patterns. Clin. Exp. Allergy.

[48] Marzano A.V., Ortega-Loayza A.G., Heath M., Morse D., Genovese G., Cugno M. (2019). Mechanisms of Inflammation in Neutrophil-Mediated Skin Diseases. Front. Immunol..

[49] Murata K., Nakao N., Ishiuchi N., Fukui T., Katsuya N., Fukumoto W., Oka H., Yoshikawa N., Nagao T., Namera A. (2022). Four Cases of Cytokine Storm after COVID-19 Vaccination: Case Report. Front. Immunol..

[50] Cro S., Cornelius V.R., Pink A.E., Wilson R., Pushpa-Rajah A., Patel P., Abdul-Wahab A., August S., Azad J., Becher G. (2022). Anakinra for Palmoplantar Pustulosis: Results from a Randomized, Double-blind, Multicentre, Two-staged, Adaptive Placebo-controlled Trial (APRICOT)*. Br. J. Dermatol..

[51] Hüffmeier U., Wätzold M., Mohr J., Schön M.P., Mössner R. (2014). Successful Therapy with Anakinra in a Patient with Generalized Pustular Psoriasis Carrying IL36RN Mutations. Br. J. Dermatol..

[52] Bindoli S., Giollo A., Galozzi P., Doria A., Sfriso P. (2022). Hyperinflammation after Anti-SARS-CoV-2 MRNA/DNA Vaccines Successfully Treated with Anakinra: Case Series and Literature Review. Exp. Biol. Med..

[53] Lazaros G., Anastassopoulou C., Hatziantoniou S., Kalos T., Soulaidopoulos S., Lazarou E., Vlachopoulos C., Vassilopoulos D., Tsakris A., Tsioufis C. (2021). A Case Series of Acute Pericarditis Following COVID-19 Vaccination in the Context of Recent Reports from Europe and the United States. Vaccine.

